# Toward onset prevention of cognitive decline in adults with Down syndrome (the TOP-COG study): study protocol for a randomized controlled trial

**DOI:** 10.1186/1745-6215-15-202

**Published:** 2014-06-03

**Authors:** Sally-Ann Cooper, Muriel Caslake, Jonathan Evans, Angela Hassiotis, Andrew Jahoda, Alex McConnachie, Jill Morrison, Howard Ring, John Starr, Ciara Stiles, Frank Sullivan

**Affiliations:** 1 Institute of Health and Wellbeing, University of Glasgow, Mental Health and Wellbeing Unit, Gartnavel Royal Hospital, Administrative Building, 1055, Great Western Road, Glasgow G12 0XH, UK; 2 Institute of Cardiovascular and Medical Sciences, University of Glasgow, McGregor Building, 2nd floor, Western Infirmary, Glasgow G11 6NT, UK; 3 University College London, Bloomsbury Campus, Charles Bell House, 67-73 Riding House Street, London W1W 7EY, UK; 4 Robertson Centre for Biostatistics, University of Glasgow, Boyd Orr Building, Glasgow G12 8QQ, UK; 5 Institute of Health and Wellbeing, University of Glasgow, General Practice and Primary Care, 1 Horselethill Road, Glasgow G12 9LX, UK; 6 School of Clinical Medicine, University of Cambridge, Douglas House, 18b Trumpington Road, Cambridge CB2 2AH, UK; 7 Alzheimer Scotland Dementia Research Centre, 7 George Square, Edinburgh EH8 9JZ, UK; 8 Gordon F Cheesbrough Research Chair and Director of UTOPIAN, University of Toronto, North York General Hospital, 4001 Leslie Street, Toronto, ON M2K 1E1, Canada

**Keywords:** Alzheimer disease, Dementia, Down syndrome, Neuropsychology, Primary prevention, Simvastatin, Statin

## Abstract

**Background:**

Early-onset dementia is common in Down syndrome adults, who have trisomy 21. The amyloid precursor protein gene is on chromosome 21, and so is over-expressed in Down syndrome, leading to amyloid β (Aβ) over-production, a major upstream pathway leading to Alzheimer disease (AD). Statins (microsomal 3-hydroxy-3-methylglutaryl coenzyme A reductase inhibitors), have pleiotropic effects including potentially increasing brain amyloid clearance, making them plausible agents to reduce AD risk. Animal models, human observational studies, and small scale trials support this rationale, however, there are no AD primary prevention trials in Down syndrome adults. In this study we study aim to inform the design of a full-scale primary prevention trial.

**Methods/Design:**

TOP-COG is a feasibility and pilot double-blind randomized controlled trial (RCT), with a nested qualitative study, conducted in the general community. About 60 Down syndrome adults, aged ≥50 will be included. The intervention is oral simvastatin 40mg at night for 12 months, versus placebo. The primary endpoint is recruitment and retention rates. Secondary endpoints are (1) tolerability and safety; (2) detection of the most sensitive neurocognitive instruments; (3) perceptions of Down syndrome adults and caregivers on whether to participate, and assessment experiences; (4) distributions of cognitive decline, adaptive behavior, general health/quality of life, service use, caregiver strain, and sample size implications; (5) whether Aβ42/Aβ40 is a cognitive decline biomarker. We will describe percentages recruited from each source, the number of contacts to achieve this, plus recruitment rate by general population size. We will calculate summary statistics with 90% confidence limits where appropriate, for each study outcome as a whole, by treatment group and in relation to baseline age, cognitive function, cholesterol and other characteristics. Changes over time will be summarized graphically. The sample size for a definitive RCT will be estimated under alternative assumptions.

**Discussion:**

This study is important, as AD is a major problem for Down syndrome adults, for whom there are currently no effective preventions or treatments. It will also delineate the most suitable assessment instruments for this population. Recruitment of intellectually disabled adults is notoriously difficult, and we shall provide valuable information on this, informing future studies.

**Trial registration:**

Current Controlled Trials ISRCTN Register ID: ISRCTN67338640 (17 November 2011)

## Background

### Down syndrome and dementia

Adults with Down syndrome have a high prevalence of dementia of Alzheimer disease (AD) type from middle age onward [1,2]. Dementia is a highly disabling disorder that results in progressive deterioration, increasing dependency as well as health and social support resource consumption and, ultimately, premature death. It has an impact on adults with dementia and their family and friends and also is a source of major societal and economic costs. Down syndrome is the commonest cause of early-onset dementia, with 40% of adults with Down syndrome ages 50 years and older acquiring it [1,2]. Almost everyone with Down syndrome ages 40 and older has neuropathological changes due to AD [3]. Because the life expectancy of people with Down syndrome has increased rapidly, with the majority now living beyond 50 years of age, preventive measures against AD are urgently needed.

The amyloid precursor protein (APP) gene is located on chromosome 21 and thus is overexpressed in Down syndrome (trisomy 21), leading to amyloid β (Aβ) overproduction. Excess Aβ levels form insoluble plaques and are a major upstream pathway leading to AD [4]. This is thought to be the mechanism of action resulting in the very high rates of AD in adults with Down syndrome. The pathology of dementia in adults with Down syndrome differs from that in the general population. In the general population vascular dementia and dementia in AD often cannot be clinically differentiated. Indeed, an increasing body of knowledge suggests that vascular changes and AD pathology are interrelated [5,6]. In contrast, adults with Down syndrome develop a relatively “pure” form of AD that supports the “amyloid hypothesis” in the general population. This is so because people with Down syndrome have a remarkable resilience to atherosclerosis [7,8], possibly due to the cystathionine β synthase gene’s location on chromosome 21, which is overexpressed in Down syndrome. This leads to decreased homocysteine levels and thus reduced arteriosclerosis. As Down syndrome adults are atheroma-free [8], with low blood pressure [8], and have low vascular dementia rates, their dementia is a “pure” model of dementia in AD, unlike that in the general population. In view of the specific genetic differences in people with Down syndrome, the findings produced by trials in the general population cannot be extended to people with Down syndrome. There are currently no effective interventions to prevent dementia onset in routine clinical practice, but proof of concept has been established in older adults with Down syndrome [9].

### Evidence to date on a role for statins in Alzheimer disease

Normally, Aβ production is balanced by Aβ clearance via apolipoprotein E (ApoE) receptors and the low-density lipoprotein receptor (LDLR) [10]. LDL is bound by LDLR and taken into the cell ending where it is degraded, and cholesterol is made available for repression of microsomal 3-hydroxy-3-methylglutaryl coenzyme A (HMG CoA) reductase, which is the rate-limiting step in cholesterol synthesis. Statins are HMG CoA reductase inhibitors and thus are plausible agents to use to reduce AD risk. Supporting evidence has been produced by experimental studies, observations of populations, case-controlled observational studies, prospective cohort studies, secondary prevention trials and small-scale primary prevention trials with high-risk groups, as described in the following paragraphs.

The brain has high levels of cholesterol. Cholesterol is synthesized locally, and its elimination utilizes ApoE. Its synthesis modulates the production of Aβ. Individuals with the ApoE e4/4 allele are at particularly high risk for developing AD in both the general population and the population with Down syndrome, highlighting the role of Aβ. In the general population without dementia, ApoE e4 is associated with relative cognitive decline at age 79 years [11], but healthier lipid profiles, such as higher erythrocyte ω-3 polyunsaturated fatty acid content, are significant only in the absence of the ApoE e4 allele [12]. The hypothesized pathway link between lipid metabolism and AD is amyloid clearance by the LDLR family of proteins. Brain LDLR activity has been shown to be increased significantly, especially in astrocytes, by statin treatment [13]. The results of studies with experimental animal models have demonstrated that LDLR deficiency causes hypercholesterolemia, cerebral β-amyloidosis and learning deficits [14] and that statins improve learning and slow AD pathology development [15]. LDLR-deficient Tg2576 mice develop hypercholesterolemia and age-dependent cerebral β-amyloidosis [14]. In the study by Cao *et al*. [14], LDLR-deficient Tg2576 mice showed more spatial learning deficits than LDLR-intact Tg2576 mice did after the manifestation of Aβ deposition. Although LDLR genotypes did not affect the expression level of the Aβ precursor protein transgene, there was a significant increase in Aβ deposition accompanied by an increase of *APOE* expression in LDLR-deficient Tg2576 mice.

In humans, almost all the evidence to date is drawn from the general population rather than from people with Down syndrome. Rates of dementia in AD appear to be low in populations with low blood cholesterol levels and diets low in fat and cholesterol [16,17]. Some study researchers have reported that high cholesterol levels increase the risk of AD [18], although not all found this to be so [19]. However, brain cholesterol is synthesized locally, and it is unclear whether blood levels are a suitable proxy measure.

Researchers in several case–control studies have reported a lower risk of dementia among statin users than among controls [20-26]. As highlighted in a recent Cochrane review [27], the earlier studies were challenged on indication bias. However, the finding in these studies has been replicated, despite recent improvements in access to health care for people with dementia.

In several prospective cohort studies, including recent well-conducted, large-scale studies, investigators reported that the use of statins predicted reduced incidence of AD or was associated with trends toward slower cognitive decline [26,28-33] or reduced risk of hospitalization due to dementia [34]. The strength of association between statin use and reduction in incidence of AD has been shown to diminish with age [32]. Other study researchers have reported no associations of statin use with AD [25,35], but not all differentiated dementia in AD from vascular dementias. Additionally, the amyloid pathway may not be the major disease determinant in some cases. For example, the religious order study participants had an exceptionally high mean educational level of 18.2 years [36]. Also, as amyloid pathway irregularities are upstream of dementia development, the period of observation of statin treatment may not cover all of the at-risk period. Researchers in one study found that lifelong cognitive change data found statins did indeed protect against cognitive decline in a population at approximately 80 years age [37].

Only one study has been conducted on the use of statins in adults with Down syndrome. In it, the investigators studied the relationship between statin use and incident dementia in AD over the course of 5 years in a prospective US cohort of 123 participants ages 40 years and older [9]. The participants on statins had less than half the risk of incident dementia. In the same cohort, persons with measurements of Aβ42 in the middle or highest range were found to be more than twice as likely to have incident dementia and persons with Aβ levels in the highest third were more likely to die [38]. To the best of our knowledge, to date, no trials of statins with adults with Down syndrome have been reported and none are currently registered.

With regard to trials involving the general population, authors of a recent Cochrane review found only two published statin RCTs for the primary prevention of dementia [27]: the MRC/BHF Heart Protection Study (HPS) [39] and the Prospective Study of Pravastatin in the Elderly at Risk (PROSPER) study [40]. The HPS investigators used brief telephone interviews to assess cognition and found no effect of simvastatin 40 mg OD versus placebo over the course of 5 years [39]. In the PROSPER study, which was of older people, the trialists did not report any benefits, on the basis of cognitive testing, from pravastatin 40 mg OD therapy over a mean course of 3.2 years [40]. However, their conclusions are not relevant to adults with Down syndrome, as the study participants were selected specifically for having vascular disease and/or vascular risk factors and not for Aβ overproduction. Also, in neither trial did the investigators study incident dementia in AD specifically, and cognitive measures were only secondary outcomes. Indeed, the HPS team did not collect baseline cognitive data, and good baseline cognitive ability was required in the PROSPER study (Mini Mental State Examination score >24). Researchers in smaller studies including “AD high-risk groups” have found benefits at 4 months and 6 months [41,42]. The investigators in an AD high-risk study, the European/Australasian Stroke Prevention in Reversible Ischaemia Trial (ESPRIT) study, recruited 100 people ages 35 to 69 years who had a parent with AD and studied simvastatin 80 mg OD. After 9 months, no difference was found in change in cerebrospinal fluid (CSF) Aβ42, although the degree of change was influenced by the participant’s underlying risk profile [43].

In a small, secondary prevention randomized controlled trial (RCT), the investigators randomized 63 people with mild to moderate dementia in AD to atorvastatin 80 mg once daily (OD) or placebo and demonstrated slowing in cognitive decline in the statin group at 6 and 12 months [44]. In a larger-scale, double-blind, placebo-controlled study (the LEADe study), researchers recruited 640 participants with mild to moderate dementia and tested the addition of atorvastatin 80 mg to treatment with a cholinesterase inhibitor. They found no difference in change in cognition between the intervention and control groups after 72 weeks [45]. Two large, placebo-controlled, secondary prevention trials are in progress: the statins/CLASP (Collaborative Low-dose Aspirin Study in Pregnancy) study (investigating simvastatin 40 mg) and the UCSD Statin Study (investigating simvastatin 20 mg or pravastatin 40 mg). However, statins are theoretically more effective in primary than in secondary prevention, so the results of these studies will be less relevant to the study outlined in this protocol. Authors of two more reviews recently concluded that trials are indicated specifically when AD is due to amyloid overproduction [46,47]. This is exactly the situation in Down syndrome. Authors of a recent systematic review of statins and cognitive function found there is an absence of well-powered RCTs for most cognitive outcomes and concluded that larger and better-designed studies are needed [48].

Much of the literature on aging and dementia in the general population does not apply to the atheroma-free Down syndrome population. Given the exceedingly high prevalence of dementia in AD in the Down syndrome population, it is crucially important that trials with and for them be undertaken.

### Choice of statin and dose

There is no Down syndrome literature to inform the choice of statin to investigate in a primary prevention study or to confirm whether its safety profile is similar to that in the general population. We therefore selected simvastatin on theoretical rather than empirical grounds for the following reasons:

1. Simvastatin is more lipophilic than hydrophilic compared with other statins [49]; therefore, it crosses the blood–brain barrier more easily and hence is expected to be more effective than other statins.

2. Simvastatin has a good safety record in the general population. Researchers in clinical trials have reported that myalgia occurred in 1.2% of participants who received 40 mg OD [50]. They also found no difference in muscle pain or weakness between participants treated with simvastatin 40 mg OD or placebo for 5 years or in the number who discontinued treatment due to musculoskeletal problems [39]. Adverse reactions increase at higher doses [51]; hence we chose to use 40 mg OD in our present study. The incidence of fatal rhabdomyolysis has been estimated at 0.12% per 1 million prescriptions on the basis of data derived from the US Food and Drug Administration databases and the National Prescription Audit Plus [50].

3. There is evidence derived from a general population pilot RCT to support our choice of simvastatin 40 mg OD. After 26 weeks, participants with mild, but not severe, dementia in AD taking simvastatin 80 mg OD compared with placebo had statistically significantly higher scores on the Mini Mental State Examination and significantly decreased CSF Aβ40 levels. The reduction was correlated with CSF reduction in the cholesterol metabolite 24S-hydroxycholesterol. In participants with severe dementia, the large proportion of Aβ deposited in amyloid plaques may explain why a reduction was not detected in CSF [52]. Additionally, 57 participants at high risk for AD showed improvements in verbal fluency and on working memory measures in a 4-month, double-blind RCT of simvastatin 40 mg OD [41].

### Neuropsychological test instruments

High dementia incidence and caregiver-reported adaptive functional decline over time (that is, a proxy measure of decline) are well-reported in adults with Down syndrome. Neuropsychological test materials used specifically to measure cognitive decline necessarily must differ from the tests used with the general population, as adults with Down syndrome have preexisting cognitive deficits and therefore existing norms do not apply. Furthermore, when assessing longitudinal decline, floor effects on general population measures prevent change being registered. Researchers have attempted to phenotype the early stages and progression of dementia in the Down syndrome population with adapted or specially devised assessments [2,53-57]. Investigators in small-scale studies have found a pattern of deterioration similar to that in the general population, with memory problems being the first clinical marker [53,54]. However, recently, study researchers have suggested that deficits in executive function, characterized by planning problems, personality changes and development of problem behaviors, might predate other aspects [2,57,58] and stem from the frontal lobe problems associated with Down syndrome [2]. These studies are limited by small sample sizes. Researchers in several studies who have utilized a range of measures, principally in the domains of memory, attention and executive function, have investigated cognitive decline with aging in people with Down syndrome [53,56,59,60]. However, to the best of our knowledge, no longitudinal reports of more than a handful of participants with Down syndrome ages 50 years and older have been published. Although researchers who studied 322 adults with intellectual disabilities published normative data derived from the Neuropsychological Assessment of Dementia in Intellectual Disabilities (NADIID), their investigation included few people with Down syndrome 50 years of age and older, and very sparse published longitudinal data on cognitive decline in people in this age group [53].

We anticipate that performance on standardized tests of cognitive function will be a more accurate and sensitive measure of decline than caregiver-reported changes in adaptive function. Hence, phenotyping is important. In this study, we will delineate the instruments most sensitive for detecting change by assessing the distribution of scores cross-sectionally and over time in relation to age, baseline scores and other participant characteristics. Hence the results of this study will enable the development of a new battery of tools comprising those with the greatest utility for the early detection of decline. This is important in the planning of a full-scale, primary prevention RCT.

### Study aims

The aims of the study are (1) to acquire data to design a full-scale multi-center RCT of simvastatin for the primary prevention of dementia in AD, (2) to test recruitment and retention strategies to inform future trials with this population, (3) to determine the best instruments to use in future studies measuring cognitive decline in adults with Down syndrome and (4) to investigate mechanisms, using Aβ42/Aβ40 measurements as a putative surrogate biological marker. Additionally, consent will be obtained for subsequent future longer-term follow-up by record linkage to routinely collected health data and for samples to be banked at the University of Glasgow for potential future research.

## Methods/Design

### Type of study

**Figure 1:**
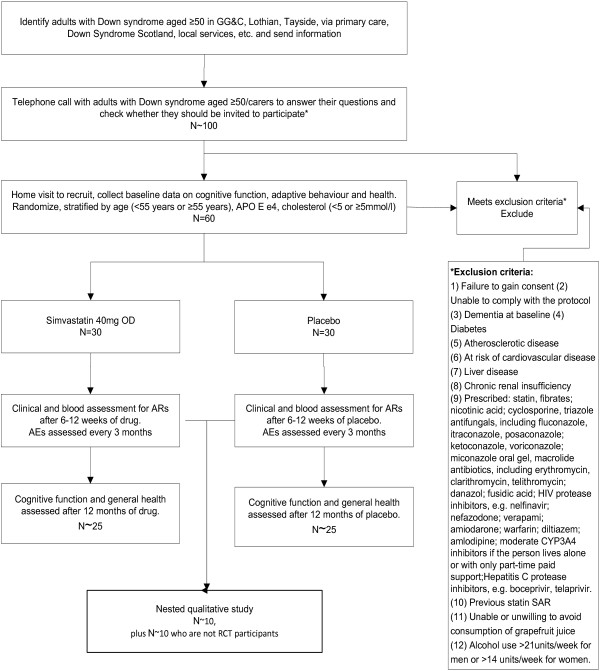
**Flowchart of the study protocol.** AE, Adverse event; APO E, apolipoprotein E; AR, Adverse reaction; CYP3A4, Cytochrome P450 3A4; GG&C, NHS Greater Glasgow & Clyde; OD, Once daily; RCT, Randomized controlled trial; SAR, Serious adverse reaction.

The study is a double-blind RCT of 12 months of simvastatin 40 mg OD by mouth versus placebo. It includes a nested qualitative study. The flowchart shown in Figure 1 summarizes the study protocol.

1. The study will be randomized to determine if, in this population, participants and caregivers are willing to receive the intervention or placebo without knowing which is being administered. Randomization will be stratified for baseline characteristics known to influence cognitive decline (age <55 and ≥55 years and ApoE e4 genotype) or characteristicswhere there is some limited evidence of influence (cholesterol <5 mmol/L and ≥5 mmol/L) to prevent any imbalance in participant types between treatment groups.

2. Semistructured interviews will be conducted with participant-caregiver dyads to gain an understanding of their views on their decision whether to participate and be randomized, and their assessment experiences.

### Research questions

The research questions we will seek to answer are listed below.

1. What are the trial recruitment and retention rates and recruitment sources?

2. What are the rates of tolerability and safety of simvastatin 40 mg OD?

3. Which instruments are the most sensitive for detecting early cognitive decline with the least floor effect in adults with Down syndrome adults?

4. What are the perceptions of adults with Down syndrome and their caregivers regarding their decision whether to participate and be randomized and their assessment experiences?

5. What are the distributions of the primary outcome measure (cognitive decline) and key secondary outcome measures (adaptive behavior, general health and quality of life, service use and caregiver strain) that would be used in a definitive RCT, and what are the sample size implications of these distributions?

6. Is Aβ42/Aβ40 a biomarker for cognitive decline?

7. Do the results support proceeding to a full RCT?

The outcome measures we will use to answer each of the research questions listed above are described in the subsections that follow.

#### Research question 1: feasibility

1. The numbers screened and recruited each month over the course of the recruitment period

2. A measure of the retention of participants in the study after 12 months

3. The percentage of the total number of participants recruited from each source and the number of contacts with each source to achieve this (The source will be identified during the initial telephone call to assess suitability of inviting the person to participate. Each contact made will be recorded by the researcher and Scottish Primary Care Research Network staff documenting each contact on a contact recording sheet.)

4. The number of participants recruited per base general population size.

#### Research question 2: tolerability and safety

1. Compliance will be assessed by counting returned tablets every 3 months.

2. Blood will be taken to measure muscle enzyme levels 6 to 12 weeks after starting the simvastatin or placebo treatments.

3. Interviews will be conducted every 3 months, in addition to recording spontaneously reported adverse events (AEs) using the standard sponsor’s AE standard operating procedure.

#### Research question 3: identification of the most suitable cognitive measures

On the basis of published data regarding measures of cognitive decline in people with Down syndrome, we have identified eight tests considered most likely to be sensitive to change. The tests are in the domains of memory, attention and executive function:

1. Memory for Objects from the NADIID battery [53]

2. Selective Attention Cancellation Task [60]

3. Pattern Recognition Memory from the Cambridge Neuropsychological Test Automated Battery [61]

4. Cats and Dogs test [56]

5. Tower of London Test (a test of frontal lobe executive function recently adapted for adults with intellectual disabilities by our group) [62]

6. Cued Recall Test [63]

7. Category fluency [56]

8. Story recall (adapted from the Rivermead Behavioural Memory Test for Children [64])

Scores at baseline and after 12 months of simvastatin or placebo treatment will be compared for each of these measures to identify which show the greatest degree of change over the period and have the greatest utility across the full range of intellectual disabilities.

#### Research question 4: participant/caregiver perceptions

Key themes will be identified from the semistructured interviews in the nested qualitative study to improve our understanding of participants’ perspectives about research participation and randomization, and their assessment experiences. The analysis will be guided by the framework approach [65].

#### Research question 5: effect size

The measures described below will be used with the adults with Down syndrome at baseline and after 12 months of simvastatin or placebo treatment to determine the effect size by comparing the simvastatin group with the placebo group.

The primary outcome measure will be cognitive decline (using the instruments outlined above) [53,60-64]. The secondary outcomes will be measured using the following instruments:

1. American Association on Intellectual and Developmental Disabilities Adaptive Behavior Scale (to measure the adaptive function of persons with intellectual disabilities) [66]

2. Townsend’s Disability Scale (to measure general health) [67]

3. EQ-5D (the EuroQol 5-Dimension Questionnaire; to measure health outcomes and quality of life) (recently reviewed for use with people with intellectual disabilities [68])

4. Client Service Receipt Inventory (to measure demographics and extent of service use as well as social changes, such as changes in level of paid support, move to a nursing home or loss of day placement) (This instrument was designed for use with adults with intellectual disabilities and adults with mental illness.) [69,70]

5. The 12-item General Health Questionnaire (to be given to the caregivers at baseline and after 12 months to compare the health and carer strain of caregivers of participants in the simvastatin group with caregivers of participants in the placebo group) [71].

#### Research question 6: biomarkers

At baseline and after 12 months of simvastatin or placebo treatment, blood will be taken to measure Aβ40/Aβ42 levels for comparison between the simvastatin and placebo groups.

#### Research question 7: recommendations for a full randomized controlled trial

1. The size of the general population pool needed to recruit adults with Down syndrome for the full RCT will be determined on the basis of the rates of recruitment and study completion in this pilot study, combined with the treatment effect estimate and uncertainty regarding the estimate. Additionally, the sensitivity of the neuropsychological measures will inform this decision, in particular with regard to the floor effect with persons with the most severe intellectual disabilities; in other words, to provide information about the proportion of the recruited population for whom it was possible to detect and measure change and therefore the likely proportion who would need to be excluded based on their baseline assessment of intellectual function.

2. This pilot study will consider the size of the geographical recruiting area required and hence the associated costs. The full RCT would not be appropriate if there were unexpected adverse safety effects identified in this pilot study, which we consider to be unlikely.

### Ethical considerations and consent

The study was given a favorable opinion by the Scotland A Research Ethics Committee (REC). In keeping with this response, each potential participant’s capacity to decide whether to participate in the trial will be assessed through discussion of the study, which will be facilitated with the person’s caregiver. This discussion with each potential participant will include going through the information sheet, which will be designed for persons with intellectual disabilities. The discussion will include what their participation will involve and will inform them that they will not benefit personally from taking part and that they do not have to participate if they do not wish to. Any questions that the person or caregiver has will be discussed and answered. To assess the person’s ability to understand, retain and weigh information about whether to participate, the person with Down syndrome will be asked to say what they think the study is about and what it will involve if they take part. Individual informed consent will then be obtained from persons who demonstrate that they have a good grasp of the study and understand that they can choose whether to take part. For persons considered not to have full decision-making capacity to consent to participate in the trial, the informed consent of their legal representative, as defined in the Medicines for Human Use (Clinical Trials) Regulations 2004 (CTR), will be sought. A separate information sheet will be used with relatives and other legal representatives for this purpose. Participation in the qualitative component of the study will require a separate consent. The legal requirement for this in Scotland is to adhere to the Adults with Incapacity (Scotland) Act, 2000. Hence, capacity to consent is assessed as outlined above. Persons then consent for themselves if they have the decision-making capacity to do so; otherwise, consent is obtained from their welfare guardian or nearest relative (as defined in the Act).

### Statistical power

Approximately 60 participants with Down syndrome ages 50 and older will be randomized. Recruitment feasibility will be assessed on the bases of the number of people identified per 10,000 population and the percentages of those contacted who would like to participate, and, among those, the percentage who are eligible to participate. For example, if 200 individuals are contacted and 100 agree to be screened, from among whom 60 are eligible for randomization, then the overall recruitment rate will be 30% with a 90% confidence interval (CI) of 25% to 36%. This will be sufficiently accurate to allow planning for a larger RCT. Similarly, if 50 participants complete the 12-month follow-up, the retention rate will be 83% with a 90% CI of 73% to 91%.

The variance of the rate of cognitive decline will be estimated to calculate the sample size required for a definitive RCT. The precision of this estimate is a function of the sample size in this pilot study. We think that a sample size of 50 participants from whom we gather 12-month outcome data is appropriate at this stage to provide a variance estimate that is reasonably precise, without requiring that we recruit an excessively large sample. With 50 participants, a 90% CI for the variance in the rate of cognitive decline will have a width of approximately 70% of the estimated variance. A smaller sample size in this pilot study would greatly increase the uncertainty in the variance estimate, whereas calculation of a more precise estimate could require considerably more participants. For example, halving the width of the 90% CI for the estimated variance would require 180 participants, which we believe is too large for a pilot study designed to show the feasibility of an RCT.

### Sample size for the qualitative study

We do not know in advance how many interviews will be necessary to reach saturation (with no new themes emerging), but we will plan to interview ten dyads of participants with Down syndrome and their caregivers and will also attempt to recruit ten dyads of adults with Down syndrome and their caregivers who choose not to participate in the pilot RCT. It is possible that some dyads will agree to this single, semistructured interview despite having declined to participate in the pilot RCT.

### Participants and their recruitment

For the RCT, we will recruit approximately 60 adults with Down syndrome ages 50 and older. Potential participants will be given or sent information packs and invited to reply to the research team if they are interested in further information about participating.

Although all older adults with Down syndrome will be known to their primary care teams, it is likely that a more efficient recruitment strategy will combine primary care, registry-based and wider recruitment methods, with snow-balling. We will test recruitment using a multipronged approach in Greater Glasgow and Clyde, Lothian and Tayside utilizing the following resources:

1. Scottish Primary Care Research Network

2. Scottish Dementia Clinical Research Network

3. Down Syndrome Scotland membership list

4. Scottish Consortium for Learning Disabilities and their e-SAY project

5. Professionals working within specialist intellectual disabilities health and social work services

6. Larger provider organizations of 24-hour support packages and specialist day care

If the recruitment rate is lower than anticipated within the first 2 months of recruitment initiation, then approvals will be sought to expand recruitment efforts into other areas of Scotland utilizing the same methods. If interest to participate in the study is high, we will recruit the first 60 suitable participants while continuing to measure the response rate. To judge the most effective means of recruitment, during the screening call to gauge each individual’s eligibility to participate in the study, we will inquire if the person knows the source of the information pack they received which resulted in their replying for more information about the study.

Two groups of participants will be selected for the qualitative study. They will be drawn from among (1) those who participated in the RCT and (2) those who declined to participate in the RCT or did not respond to the invitation to participate. For the first group, we will try to avoid bias by purposively sampling ten participants and their caregivers (including people across the range of ability levels of intellectual disabilities and paid and family caregivers) who had baseline assessments during the first 3 months of recruitment (maximum variance sampling). For the second group, we will also attempt to recruit ten Down syndrome and caregiver dyads who did not take up the invitation to participate in the pilot RCT. We will attempt to recruit people with family caregivers, people with paid caregivers and people across the range of ability levels of people with intellectual disabilities, but we acknowledge that there may in limitations in how successful we will be with this second group.

### Inclusion and exclusion criteria

The inclusion criteria are (1) a diagnosis of Down syndrome and (2) age 50 years or older. The exclusion criteria are listed below:

1. No consent obtained

2. Unable to comply with the protocol, including providing blood or saliva for baseline ApoE e4 measurement and venous or capillary blood for cholesterol measurement

3. Dementia at baseline (as the study is investigating primary prevention)

4. Diabetes (as this is an indication for prescription of a statin)

5. Clinically evident atherosclerotic disease (as this is an indication for prescription of a statin)

6. Being at risk for cardiovascular disease (as this is an indication for prescription of a statin)

7. Liver disease

8. Chronic renal insufficiency

9. Currently being prescribed any of the following:

a. A statin

b. Fibrates

c. Nicotinic acid

d. Cyclosporine

e. Triazole antifungals, including fluconazole, itraconazole, posaconazole, ketoconazole, voriconazole, miconazole oral gel, verapamil)

f. Macrolide antibiotics (including erythromycin, clarithromycin, telithromycin)

g. Danazol

h. Fusidic acid

i. HIV protease inhibitors (for example, nelfinavir, nefazodone, verapamil, amiodarone, warfarin, diltiazem, amlodipine)

j. Moderate CYP3A4 inhibitors (if the person lives alone or has only part-time paid support)

k. Hepatitis C protease inhibitors (for example, boceprevir, telaprevir)

10. Previous serious adverse reaction to a statin

11. Unable or unwilling to avoid consumption of grapefruit juice

12. Excessive alcohol use (defined as >21 U/wk for men and >14 U/wk for women)

### Group allocation and blinding

Participants will be randomly assigned to either simvastatin or placebo and stratified by age, ApoE e4 allele and cholesterol level. After collecting baseline data and samples, the researcher will notify the Robertson Centre for Biostatistics (RCB) of the participant’s study number and age via the web portal. The laboratory will notify the RCB of the participant’s ApoE status and cholesterol level, also via the web portal. The RCB will then notify the pharmacy of group allocation and generate an email to the research assistant notifying him or her that randomization has taken place as well as of the medication pack number assigned. The pharmacy will then dispense the medication. A verifiable audit trail will be ensured. The research assistant will telephone participants to check whether the medication has arrived and instructions are fully understood. The research team will therefore remain blinded to both ApoE and group allocation status, as will the participants and their caregivers. Medications will be dispensed within 4 weeks of baseline data collection. This time frame will allow for batching of the ApoE analyses.

### Duration of participation

#### Participation

Each participant’s participation in the study will last for 12 months postrandomization unless they withdraw prematurely.

#### Completion

The date of completion for safety is defined as the last dose of simvastatin or placebo plus 30 days. For other study outcomes, each participant will be considered to have completed the study either after the completion of the last assessment visit or after receiving the last dose of simvastatin or placebo, whichever is later. The date of discontinuation will be recorded as the date on which a participant and/or investigator determines that the participant can no longer comply with the requirements for any further study visits or assessments.

### Assessments

Assessments and blood tests will be conducted either in the person’s own home or at another venue if the participant/caregiver prefers. Home visits will be offered in order to increase recruitment and retention and for the convenience of the participants and caregivers, as well as to conduct the psychometric tests as accurately as possible by conducting them in a familiar, comfortable environment for the participant.

### Intervention

The intervention is simvastatin 40 mg at night by oral administration. The simvastatin will be overencapsulated. The control group will receive an oral placebo capsule at night by mouth. The capsules are to be swallowed whole, not chewed. Dose modifications are not allowed. A summary of product characteristics is available, as is an Investigational Medicinal Product Dossier. The drug and placebo will be prepared by the Pharmacy Production Unit based at the Western Infirmary in Glasgow.

#### Unblinding procedure

Unblinding will be permitted in emergencies where, for medical or safety reasons, it is necessary to know which treatment a participant has received. Study participants will be provided with a Participant Alert Card that will include the name of the investigational study drug, their study number, the investigator’s name and a 24-hour telephone number for unblinding purposes. Unblinding will be done via a telephone menu system. Several prompts in the system will warn the user that he or she is required to be a health professional and that name and other pertinent information must be recorded. At each unblinding, an email alert to the Chief Investigator will be generated. Requests will be set at a maximum of two per 24 hours to prevent malicious unblinding. The Participant Alert Card will be collected from participants at the end of their involvement in the study.

#### Expected adverse reactions

The expected adverse reactions are listed below. There is no theoretical reason to expect a higher rate in adults with Down syndrome.

1. Myalgia

2. Myositis

3. Rhabdomyolysis

4. Flulike symptoms

5. Fatigue

6. Headache

7. Nausea

8. Diarrhea

9. Fatal and nonfatal hepatic failure

10. Raised HbA1c and fasting glucose

11. Diabetes mellitus

12. Cognitive impairment (rarely)

#### Definitions of muscular adverse reactions

1. Alanine aminotransferase and aspartate aminotransferase elevation will be defined as more than three times the upper limit of normal.

2. Myalgia is defined as muscle ache or weakness without creatine kinase (CK) elevation.

3. Myositis is defined as muscular symptoms with a CK level more than ten times the upper limit of normal.

4. Rhabdomyolysis is defined as a CK level >10,000 U/L with or without muscular symptoms.

All AEs and intercurrent illnesses will be recorded, notified, reported, analyzed and managed in accordance with the CTR. The research sponsor’s standard operating procedures for recording and reporting AEs and serious AEs will be followed. An annual safety report will be submitted to the Medicine and Healthcare Products Regulatory Agency and the REC within 60 days of the anniversary of the issue of the clinical trial’s authorization. Safety data will be measured through three monthly telephone interviews.

### Data analysis

#### Recruitment

During the initial phone call, we will identify the source to which the person responded. We will describe the percentage of the total recruited from each source and the number of contacts with each source required to achieve this total (via diary records). We will also calculate recruitment by base general population size.

#### Pilot randomized controlled trial

A detailed statistical analysis plan will be prepared prior to the unblinding to treatment allocations, according to the RCB/Glasgow Clinical Trials Unit standard operating procedures. Briefly, summary statistics will be presented, with 90% confidence limits where appropriate, for each study outcome as a whole, by treatment group and in relation to baseline participant characteristics. Changes in outcomes over time will be summarized and presented graphically. The treatment effect estimate is of interest, not so much in terms of the magnitude or statistical significance of effect (the sample size is too small to draw definitive conclusions in this study), but rather regarding the uncertainty in this estimate or the variability in the rate of cognitive decline from which this estimate is derived. It is this variability estimate that will inform the sample size for a definitive trial, along with information about likely recruitment and retention rates. Statistical results will be interpreted in terms of the implications for a definitive RCT; for example, the sample size (and cost) required for screening and randomization will be estimated under alternative assumptions based on the pilot data. In additional analyses, we will examine outcomes of cognitive decline in relation to age, ApoE status, baseline cognitive function, cholesterol level and other baseline characteristics, which may provide information useful in defining the target population in future studies. We intend to apply exploratory factor analysis and other data reduction methods to examine interrelationships between different cognitive measures and their changes over time. The data gathered will not be sufficient to propose alternative outcome measures based on this analysis, but we may be able to propose novel hypotheses to be tested in a larger study.

#### Qualitative study

Topic guides will be used in the semistructured interviews, but they will be flexible. They will focus on the issues that potential study participants consider when deciding whether to take part in the study and, after the study, what made taking part a positive or negative experience for them. The interviews will be tape-recorded and transcribed verbatim. The analysis will be guided by the framework approach [63]. As such, the analytic process will be deductive in that it will be guided by the aims of the study, but it will simultaneously be inductive and flexible and thereby allow key themes to emerge. The tape-recorded interviews will be transcribed as close to the time of the interview as possible, and the researcher’s field notes will be checked to ensure accurate transcription. Analysis will begin during the interview period so that any themes which arise can be verified in later interviews. The researcher will review the transcripts to identify the key issues of importance for participant-caregiver dyads. The first four transcripts will be analyzed independently by a second member of the research team who is blinded to the themes identified by the researcher to ensure consistency of identification of themes and that the identified themes are an accurate reflection of the material. The results will be fed back to the participant-caregiver dyads to find out if they concur with our findings. The themes will be used to make suggestions about how to revise and/or improve the study processes for participants.

## Discussion

We think this study is important, as dementia is such a major problem for adults with Down syndrome. At present, there are no interventions that have been found to be effective for them in either preventing dementia or treating it. This feasibility/pilot study is the first step toward informing a large-scale RCT to determine whether simvastatin is effective in preventing or delaying the onset of dementia in this group. Additionally, the findings are likely to be relevant to clinical practice as well as to future research because, at present, there is no neuropsychological test battery accepted as best practice for use with adults with Down syndrome. This study will provide information on the instruments that may be useful in this regard. In view of the age range of the participants (50 years and over), the study should be more informative about neuropsychological test instruments than previous work with younger adults with Down syndrome. The study will allow us to determine whether our battery of cognitive tests is sensitive to cognitive decline over a 1-year period in the placebo group.

Recruitment is often a challenge in general population trials [72]. The authors of a Cochrane review on strategies to improve recruitment concluded that (1) trialists should include evaluations of their recruitment strategies and (2) funders should support these evaluations because the number of interventions that have been rigorously evaluated in the context of a real trial is low [73]. Recruitment of adults with intellectual disabilities is notoriously difficult. They are routinely excluded from general population medication trials, and very few trials have been designed specifically for them. There are challenges in conducting research with this group, and recruitment may be atypical [74,75]. In an antipsychotic drug study of adults with intellectual disabilities with aggressive behaviors, the investigators had substantial recruitment problems but excellent retention rates. Only 72% of the intended participant numbers were recruited, despite an increase in recruitment time from 2 to 4 years and an extension of study sites [75]. Previous studies of rivastigmine and donepezil with adults with Down syndrome were small in scale and of short duration and the participants recruited were adults younger than proposed in our protocol; hence they were less challenging to recruit than in our study because of the larger pool of potential participants for those studies [76,77]. As the participants in the previous studies cited already had dementia, the motivation of the participants and their caregivers might conceivably differ from that of the potential participants in our study, who will be disease-free. In a larger study of memantine, participants were recruited over a 2.5-year period in England and Norway. They included adults at the younger age of 40 years and people who did and did not have dementia; hence recruitment was less challenging than in our study [78]. The likely recruitment and retention rates with this population in the age range chosen for our study are unknown and might be atypical. There is not a body of research to understand the choices made by older persons with Down syndrome and their caregivers regarding research, as well as what motivates them whether to participate, their perceptions regarding randomization and their experiences in the studies. Concerns about what randomization is and what then happens to participants may be significant blocks to recruitment; hence an important part of this study is to better understand these perceptions with a view toward minimizing them in future studies. These knowledge gaps will be addressed in this study, which should provide valuable information on recruitment and retention to inform future studies.

## Trial status

We will complete recruitment in June 2014.

## Abbreviations

Aβ: Amyloid β; AD: Alzheimer disease; APP: Amyloid precursor protein; ApoE: Apolipoprotein E; CI: Confidence interval; CSF: Cerebrospinal fluid; CTR: Medicines for Human Use (Clinical Trials) Regulations 2004; CYP3A4: Cytochrome P450 3A4; EQ-5D: EuroQol 5-Dimension Questionnaire; HMG CoA: 3-hydroxy-3-methylglutaryl coenzyme A; HPS: Heart Protection Study; ISRCTN: International Standard Randomised Controlled Trial Number; LDLR: Low-density lipoprotein receptor; MHRA: Medicine and Healthcare Products Regulatory Agency; NADIID: Neuropsychological Assessment of Dementia in Intellectual Disabilities; RCB: Robertson Centre for Biostatistics; RCT: Randomized controlled trial; REC: Research ethics committee.

## Competing interests

The authors declare that they have no competing interests.

## Authors’ contributions

SAC was involved with the conception and design of the study, supervised data collection and wrote the funding application, the study protocol and this manuscript. MC directs the laboratory and is responsible for analyses and revised critically for intellectual content the funding application, the study protocol and this manuscript. JE was involved with the design of the neuropsychological test battery and revised critically for intellectual content the funding application, the study protocol and this manuscript. AH contributed to the study design and revised critically for intellectual content the funding application, the study protocol and this manuscript. AJ designed the neuropsychological test battery and revised critically for intellectual content the funding application, the study protocol and this manuscript. AM designed the statistical plan, was responsible for analyses and revised critically for intellectual content the funding application, the study protocol and this manuscript. JM contributed to the study design and revised critically for intellectual content the funding application, the study protocol and this manuscript. HR contributed to the study design and revised critically for intellectual content the funding application, the study protocol and this manuscript. JS was involved with the conception of the study, contribution to the study design and revised critically for intellectual content the funding application, the study protocol and this manuscript. CS acquired the study data and revised critically for intellectual content the study protocol and this manuscript. FS contributed to the study design and revised critically for intellectual content the funding application, the study protocol and this manuscript. All authors read and approved the final version of the manuscript.

## Authors’ information

SAC, BSc, MBBS, FRCPsych, MD, is a professor of learning disabilities. MC, BSc, AIMLS, FIMLS, PhD, is a professor of vascular biochemistry. JE, BSc, DipClinPsych, PhD, is a professor of applied neuropsychology. AH, MD, MA, PhD, FRCPsych, is a reader in learning disabilities. AJ, BSc, PhD, MPhil (Clin Psychol), is a professor of learning disabilities. AM, BSc, MSc, PhD, is Assistant Director of Biostatistics at the Robertson Centre for Biostatistics, University of Glasgow. JM, MBChB, MSc, PhD, DCH, FRCGP, FRCP(Edin), FRCP(Glasg), FHEA, is a professor of general practice. HR, BSc, MBBS, FRCPsych, MD, is a reader in neuropsychiatry. JS, MBBS, MA, FRCPEd, FRSPH, is a consultant in general and geriatric medicine and honorary professor of health and aging. CS, BSc, MSc, is a research assistant. FS, FRSE, FRCGP, FRCP, is a professor of research and Director of the University of Toronto Practice-Based Research Network (UTOPIAN).
